# Review of RyR1 pathway and associated pathomechanisms

**DOI:** 10.1186/s40478-016-0392-6

**Published:** 2016-11-17

**Authors:** Jessica W. Witherspoon, Katherine G. Meilleur

**Affiliations:** National Institute of Nursing Research/Tissue Injury Branch/Neuromuscular Symptoms Unit, National Institutes of Health, Bethesda, MD 20814 USA

**Keywords:** RyR1, Myopathies, Skeletal, Muscle, Oxidative, Stress, Excitation-contraction, Pathomechanism, Treatment, Mitochondria, Post-translational modifications

## Abstract

Ryanodine receptor isoform-1 (RyR1) is a major calcium channel in skeletal muscle important for excitation-contraction coupling. Mutations in the *RYR1* gene yield RyR1 protein dysfunction that manifests clinically as *RYR1*-related congenital myopathies (*RYR1*-RM) and/or malignant hyperthermia susceptibility (MHS). Individuals with *RYR1*-RM and/or MHS exhibit varying symptoms and severity. The symptoms impair quality of life and put patients at risk for early mortality, yet the cause of varying severity is not well understood. Currently, there is no Food and Drug Administration (FDA) approved treatment for *RYR1*-RM. Discovery of effective treatments is therefore critical, requiring knowledge of the RyR1 pathway. The purpose of this review is to compile work published to date on the RyR1 pathway and to implicate potential regions as targets for treatment. The RyR1 pathway is comprised of protein-protein interactions, protein-ligand interactions, and post-translational modifications, creating an activation/regulatory macromolecular complex. Given the complexity of this pathway, we divided these interactions and modifications into six regulatory groups. Three of several RyR1 interacting proteins, FK506-binding protein 12 (FKBP12), triadin, and calmodulin, were identified as playing important roles across all groups and may serve as promising target sites for treatment. Also, variability in disease severity may be influenced by prolongation or hyperactivity of post-translational modifications resulting from RyR1 dysfunction.

## Introduction

Ryanodine receptor 1-related myopathies (RyR1-RM) comprise the most common non-dystrophic congenital myopathy, with a prevalence of approximately 1/90,000 in the United States [2, 83]. Causative mutations in the gene (*RYR1*), which encode the major sarcoplasmic reticulum calcium release channel of skeletal muscle (RyR1), have been found in several myopathy subtypes. Although dominantly inherited central core disease (CCD, MIM# 11700) and recessively inherited multi-minicore disease (MmD) are the most commonly associated myopathies caused by mutations in *RYR1*, mutations have also been identified in cases of centronuclear myopathy (CNM), congenital fiber-type disproportion (CFTD), and King Denborough syndrome [41, 47, 85] These mutations result in constant calcium leak at rest, defective excitation-contraction coupling, and increased mitochondrial oxidative stress [48, 83]. Malignant hyperthermia susceptibility (MHS) trait, a dominantly inherited, severe pharmacogenetic reaction to volatile anesthetics and muscle relaxants, is an allelic condition (MIM# 145600) [85].

The clinical spectrum of *RYR1*-RM is quite broad. Even within CCD, symptoms may range from very mild to severe. Individuals with CCD typically present with proximal muscle weakness of the hip girdle, hypotonia, mild facial weakness, joint laxity and/or mild contractures, and/or orthopedic complications such as scoliosis [22, 83, 85]. Both hip girdle weakness due to fetal hypotonia and acetabular dysplasia contribute to congenital hip dislocation, which is also observed in CCD [22, 40, 45]. A more severe form of CCD is CCD-associated fetal akinesia. Related symptoms include severe hypotonia, arthrogryposis, skeletal deformities, kyphoscoliosis, and failure to thrive. In some cases, individuals survive birth, and present with strabismus and bilateral ptosis [118]. Individuals with MmD present with moderate to severe symptoms, such as proximal and distal weakness, hypotonia, a combination of contractures and/or laxity, progressive scoliosis, and, in some cases, bulbar weakness and/or external ophthalmoplegia [158]. Individuals with CNM are similar to MmD, but have more severe symptoms initially with improvement overtime. They often present with proximal muscle weakness, ophthalmoplegia, facial weakness, and respiratory impairment [82]. Symptoms associated with CFTD may include skeletal muscle wasting and weakness, hypotonia, ophthalmoplegia, ptosis, respiratory impairment, congenital hip dislocations, joint contractures, foot deformities, and kyphoscoliosis [29, 37]. In rare recessive cases, congenital onset is very severe with respiratory failure requiring ventilation [21, 22, 83, 85, 158]. The phenotype is complicated by several symptoms and may include myalgia, axial weakness, and fatigability [46, 80, 83]. Severity may vary within the family [83] with some individuals presenting with myopathy and MHS and others presenting with only MHS [85].

The defining histopathological feature on muscle biopsy is the presence of a single, central amorphous core extending longitudinally along the muscle fiber in the case of CCD or multiple smaller cores in one fiber (MmD), which in both cases are likely due to reduced mitochondrial oxidative enzyme activity as a result of mitochondrial deficiency or depletion [83, 85]. Fiber type predominance is another histological finding in RYR-RM [81, 158]. Fiber type transformation has been shown to result from mitochondrial activity relative to nitric oxide. As mentioned below in the nitrosylation section of group 5, nitric oxide binds to cytochrome C oxidase of the electron transport chain and inhibits mitochondrial respiration. This phenomenon has been shown in adipose tissue and skeletal muscle and is dependent on peroxisome proliferator-activated receptor gamma coactivator 1-alpha (PGC-1α). PGC-1α regulates muscle fiber type generation, favoring type I fibers. Additionally, PGC-1α overexpression in mouse skeletal muscle results in increased β-oxidation of fatty acids, increased muscle glucose uptake, and overexpression of proteins involved in fat oxidation and glucose transport [134]. We may better understand type I fiber predominance, and possibly uniformity, by studying PGC-1α in *RYR1*-RM skeletal muscle. PGC-1α levels may be affected greatly due to skeletal muscle fatty infiltration and mitochondrial dysfunction in *RYR1*-RM.

Type I fiber predominance and hypotrophy are identified in most cases [81, 85, 154, 158] In some cases (namely C-terminal RYR1 mutations), there is also type 1 fiber uniformity without structural changes in over 99 % of the type 1 muscle fibers. This is very similar to congenital neuromuscular disease with uniform type 1 fiber (CNMU1), and, in this disease, ophthalmoplegia is considered to be an important clinical manifestation [125, 126]. This may also be true in *RYR1*-RM where type 1 fibers are uniform in cases with ophthalmoplegia. On the other hand, cores may be absent in CNM and CFTD, where the predominant features are central nucleation and fiber-type disproportion where type 2 fibers are at least 25 % larger than type 1 fibers [154], as their names suggest. Fatty replacement, fibrosis, and/or nuclear internalization may also be present [22, 83].

In some cases, nemaline rods and cores coexist with myofibrillar disorganization. Patients with rods and cores are considered to have central core/rod disease (CCRD). Interestingly, instead of leaky RyR1 channels as noted in CCD, individuals with CCRD present with excess ryanodine receptors in the central cores [127]. In the recessive form of CCD, MmD, there is a depletion of the RyR1 protein [158].

Although RyR1 is a simple transmembrane protein (homotetrameric), the variation in symptomology in *RYR1*-RM suggests there is more to this protein and its function, including modifying factors [83, 158]. When scanning the literature, each article unveils small pieces to a bigger puzzle. This review combines several pieces to gain a more complete understanding of RyR1. Understanding RyR1 is critical for treatment development in *RYR1*-RM, especially given the lack of FDA approved treatment to date. Combined, the literature elucidates RyR1 as a simple protein with a complex pathway due to its tight regulation of ortho- and retrograde calcium flux by several factors including proteins, post-translational modifications, and ligands. Additionally, this paper highlights what is known about RyR1 mutations, the affected interaction sites, possible regulatory functions disrupted, and translation into the diseased state. These compiled results suggest target sites and regulatory complexes for potential therapies.

### RyR1 Structure

RyR1 is a major Ca^++^ ion channel in skeletal muscle. It is a six transmembrane (S1–S6) homotetrameric protein located in the sarcoplasmic reticulum and functions to release Ca^++^ from the SR to produce skeletal muscle contraction. The 3-dimensional structure of RyR1 was recently unveiled by Zalk et al. (2015). *The transmembrane region* of RyR1 is comprised of two domains including the pseudo voltage sensor domain and the pore-forming domain. S1–S4 helices form the pseudo voltage sensor domains interface with the pore-forming domain of the adjacent RyR1 subunit. S5–S6 helices and the p-segment create the pore-forming domain of RyR1. Similar to other six transmembrane ion channels, RyR1 also has a conserved glycine (aa4934). This region in the other ion channels serves as glycine hinges, allowing for the reorientation of the pore-forming regions in the ion channels. The same may hold true for RyR1. The P-segment, an extended peptide, is thought to contribute to the high conductance of RyR1 as it has an acidic predominance due to anionic amino acid residues. This is also the case for the cytosolic region of the S6 helix [156].

### RyR1 Pathway

There are several mutations in various RyR1-protein interaction and post-translational modification sites that result in autosomal dominant and recessive myopathies. *RYR1* mutations for CCD and MH are primarily located in the hot spots of RyR1. The hotspots, also referred to as domains 1–3 (D1, D2, and D3), include N-terminal residues 1–614 (sarcoplasm), central domain residues 2163–2458 (sarcoplasm), and C-terminal residues 4136–4973 (Pore-forming, SR lumen, and membrane) [155]. MH, however, does not have corresponding mutations in the pore-forming regions [146]. CCD and MH mutations result in leaky RyR1 channels. MmD, CNM, and CFTD mutations result in reduced RyR1 protein expression [10, 82]. CCRD, though uncommon, results in excess RyR1 protein [127]. The corresponding mutations for the recessive *RYR1*-RM are located across the gene [130, 144, 159].

The RyR1 pathway is comprised of several RyR1 protein-protein interactions, protein-ligand interactions, and post-translational modifications that comprise an activation/regulatory macromolecular complex. Given the complexity of this pathway, we have divided these interactions and modifications into six regulatory groups. Namely, group 1 responds to action potentials (initiation of Ca^++^ release) and changes in sarcoplasmic and sarcoplasmic reticulum [Ca^++^]. Groups 2 and 3 respond to changes in SR [Ca^++^]. Group 4 responds to changes in cAMP (elevated due to ACh release), Group 5 responds to changes in muscle O_2_ and glutathione ratio (GSH/GSSG), and group 6 seems to respond to sarcoplasmic [Ca^++^]. Each group functions to open and close the RyR1 channel and will be discussed in detail. Disease causing mutations are outlined at the end of each applicable group.

Review of 6 Regulatory Groups

- Group 1 contributes to orthograde signaling where EC coupling is initiated in response to neuromuscular stimulation.
- Group 2 includes RyR1 interdomain interactions that contribute to the opening and closing of the channel externally (group 1) and internally (group 3).
- Group 3 regulates retrograde signaling depending on SR [Ca^++^] and calsequestrin (CSQ) phosphorylation/dephosphorylation states.
- Group 4, like group 1, is activated based on neuromuscular stimulation with the exception that group 1 responds to a resulting action potential, and group 4 responds to resulting cAMP production.
- Group 5 is comprised of post-translational modifications (nitrosylation, oxidation, glutathionylation, and palmitoylation) of which all act on RyR1 cysteine residues regulating the key proteins in the other groups.
- Group 6 includes extracellular ligands that when bound stabilize the closed-state of non-voltage activated RyR1 channels until the RYR1 open state is activated by PKA-dependent phosphorylation of RyR1 (group 4). Ca^++^ has both activation and inhibitory sites on RyR1 affecting Calmodulin (CaM)-binding (group 2).

#### Group 1

**Fig. 1:**
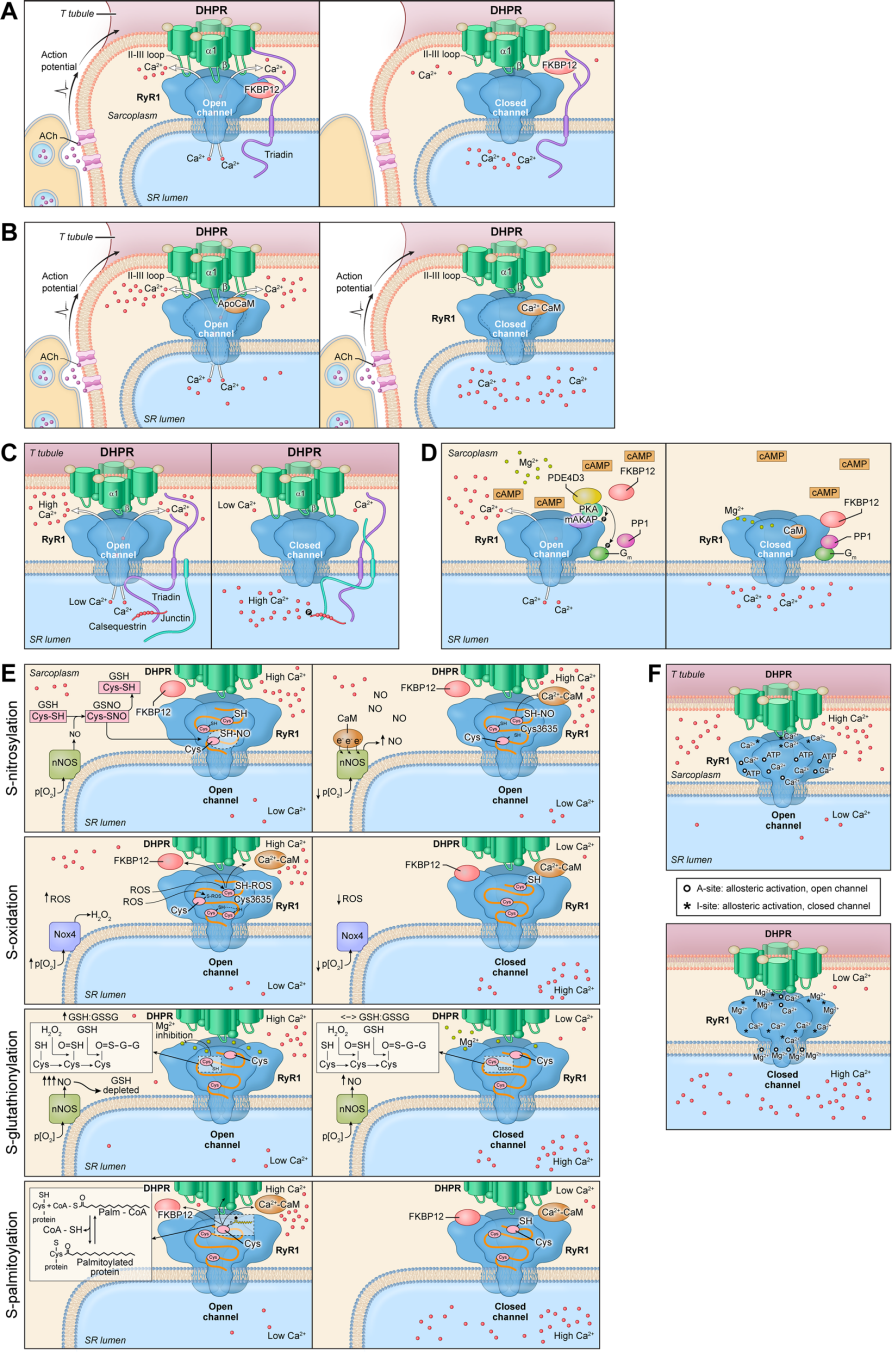
**a** *Group 1.* 1) RyR1 open (voltage-gated): RyR1 activation in response to DHPR activation by acetylcholine (ACh) release at the neuromuscular junction in skeletal muscle 2) RyR1 closed: no neuromuscular-stimulated ACh release. **b** *Group 2.* 1) RyR1 open: No interdomain interaction (unzipped) with bound ApoCaM in response to RyR1 activation preceded by DHPR activation resulting in high sarcoplasmic Ca﻿^2^^+﻿ ^2) RyR1 closed: bound Ca^2+^-Cam at high [Ca^2+^] resulting in interdomain interaction (zipped) and﻿ ﻿high sarcoplasmic reticulum (S﻿R) Ca﻿^2+^. **c** *Group 3.* 1) RyR1 open: RyR1-triadin interaction due to triadin-calsequestrin (CSQ) interaction at high Ca^2+^ bound CSQ resulting in high sarcoplasmic Ca^﻿2+^ 2) RyR1 closed: RyR1-junctin interaction due to junctin-CSQ interaction at low Ca^2+^ bound CSQ in phosphorylated state  resulting in high SR Ca﻿^2+^. **d** *Group 4.* 1) RyR1 open: RyR1 and Gm phosphorylation by PKA due to high [cAMP] re﻿sulting in high sar﻿coplasmic Ca﻿^2+ ^2) RyR1 closed: RyR1 dephosphorylation by PP1 due to low [cAMP] resulting in high SR Ca﻿^2+^. **e** *Group 5. S-nitrosylation*. 1) RyR1 open: nitrosylation of RyR1 cysteine residues by nitric oxide-glutathione reaction in response to physiologic p(O_2_)  resulting in high sarcoplasmic Ca﻿^2+^2) RyR1 open: nitrosylation of RyR1 by nitric oxide overrides Ca-CaM inhibitory effect resulting in high sarcoplasmic Ca﻿^2+^ *S-oxidation*. 1) RyR1 open: oxidation of RyR1 cysteine residues by reactive oxygen species reaction in response to increased physiologic p(O_2_) resulting in high sarcoplasmic Ca﻿^2+^ 2) RyR1 closed: no RyR1 oxidation at low physiologic p(O_2_) with bound Ca^2+^-CaM res﻿ulting in ﻿high SR Ca﻿^2+^ *S-glutathionylation*. 1) RyR1 open: initial increase in reduced glutathione (GSH) stimulating glutathionylation of RyR1 cysteine residues by GSH, in turn, decreasing the GSH:GSSG and reducing RyR1 sensitivity to Mg^2+^ inhibition resulting in high sarcoplasmic Ca﻿^2+^ 2) RyR1 closed: no glutathionylation until GSH:GSSG ratio is restored res﻿ulting in high SR Ca﻿^2+^ *S-palmitoylation*. 1) RyR1 open: palmitoylation of RyR1 cysteine residues by the fatty acid palmitoyl-CoA resulting in high sarcoplasmic Ca﻿^2+^2) RyR1 closed: no palmitoylation resulting in high SR Ca﻿^2+^. **f** *Group 6.* 1) RyR1 open: RyR1-ligand (Ca^2+^and ATP) binding activation resulting in high sarcoplasmic Ca﻿^2+^ 2) RyR1 closed: RyR1-ligand (Ca^2+^ and Mg^2+^) binding deactivation resulting in high SR Ca﻿^2+^

Group 1 (Fig. 1a) responds to neuromuscular action potentials that initiate excitation-contraction coupling. RyR1 interactions in group 1 are located in the sarcoplasm and are comprised of RyR1, DHPR, FKBP12, and triadin proteins. DHPR-RyR1, FKBP12-RyR1, and triadin-RyR1 interactions potentiate the open probability of RyR1 with voltage-gated activation. This group also responds to Ca^++^ and is thus regulated by changes in sarcoplasmic [Ca^++^]. Initially, the DHPR undergoes a conformational change, which then activates RyR1. Similarly, RyR1 undergoes a conformational change resulting in RyR1 interdomain interaction (discussed later) followed by calcium release that leads to excitation-contraction coupling. FKBP12 and triadin are important in this group for regulating the opening and closing of RyR1 following DHPR activation. In taking a closer look at these proteins, we are able to better understand their interaction and how they regulate the RyR1 channel.

### DHPR

The dihydropyridine receptor (DHPR), also referred to as CaV1.1, serves as a voltage-gated L-type calcium channel as well as a voltage sensor that is essential for excitation-contraction (EC) coupling achieved via DHPR-RyR1 interaction in skeletal muscle [31, 32, 99]. DHPR serves as a voltage sensor to neuromuscular excitation for the initiation of EC coupling (orthograde signaling), and serves as a L-type Ca^++^ channel for retrograde signaling [99]. DHPRs are located in the transverse tubule of skeletal muscle clustered in tetrads. Each tetrad interacts with every other RyR1 homotetramer [116], and is activated by sarcolemmal depolarization [31, 116]. Once activated, DHPRs undergo a conformational change that leads to RyR1-mediated Ca^++^ release [105, 116].

Amino acids 1635–2636 of RyR1 are required for orthograde and retrograde signaling, whereas amino acids 2659–3720 contribute to retrograde signaling only [98, 129]. Thr^671^-Lys^690^ region of the DHPR α1 II-III loop promotes orthograde signaling and directly binds to and induces the open state of RyR1.

DHPRs are comprised of five subunits including α1, α2, β, γ, and δ [32, 107] where the β and α1 subunits have been shown to be key players in excitation-contraction coupling [105, 112]. β and α1 subunits directly interact with RyR1 [35, 105, 112].

The β subunit guanylate kinase domain binds to the II-III loop of the α1 subunit and its C-terminus binds to RyR1 residues K^3494^-R^3502^ [35, 112]. Rebbeck [112] the β subunit functions to ensure correct positioning of the DHPR to allow α1 subunit coupling to RyR1 and maintains the open state of RyR1 in the presence of Ca^++^ and ATP. However, physiologic levels of Mg^2+^ inhibit β subunit activity [112], but the effect of Mg^2+^ is relieved by activation of the RyR1 voltage sensor, DHPR, where magnesium is disassociated from the inhibitory site on RyR1 [86, 124]. It has been shown that Ca^++^ and Mg^++^compete for the same binding site on RyR1. Unlike Mg^2+^, Ca^++^ reinforces the open state of RyR1 [86].

The II-III loop of the α1 subunit is critical for orthograde signaling to RyR1 in response to DHPR voltage-gated activation [98] and also enhances DHPR function via retrograde signaling from RyR1 to the DHPR [97, 104]. Orthograde signaling results in the release of Ca^++^ from the sarcoplasmic reticulum through the RyR1 ion channel. In contrast, RyR1-DHPR retrograde signaling has been suggested to promote inactivation of the DHPR “to limit SR Ca^++^ release and store depletion.” This hypothesis is based on the Y522S mutation, in myotubes of RyR1 knock-in mice, altering voltage-dependent inactivation of the DHPR where the voltage-dependent inactivation of DHPR-triggered Ca^++^ release was shifted to more negative holding potentials. In doing so, the voltage threshold for Ca^++^ release is lowered, limiting Ca^++^ release. Heat also results in this shift. Given the reduced Ca^++^ release, it is proposed that steady state DHPR-inactivation may be a compensatory mechanism used to counteract excessive Ca^++^ leak and SR Ca^++^ store depletion. In cases where this compensatory mechanism did not exist, the mutant (Y522S) myotubes exhibited Ca^++^ leak and SR Ca^++^ store depletion [6]. Recently, DHPR-inactivation, due to prolonged depolarization, as the primary cause of limiting excessive Ca^++^ leak and SR Ca^++^ store depletion has been challenged, and instead is believed to primarily be due to SR Ca^++^ store depletion triggering steady-state DHPR-inactivation [93]. Although controversial, investigating these mechanisms in greater detail to identify future paths toward treatment is essential.

### FKBP12

FK506-binding protein 12 (FKBP12) is encoded by the *calstabin-1* gene and is located in the sarcoplasm of skeletal muscle. There are four FKBP12 subunits that bind to the homotetrameric RyR1 protein in a 1:1 manner [151]. Until recently, FKBP12 has been shown to bind to RyR1 at aa sites 2461 and 2462 [11, 61, 65]. Further exploration of the FKBP binding sites on RyR1 revealed that FKBP interacts with the N-terminal (76–619) and central (2157–2777) domains of RyR1 [65]. More specifically, FKBP binds at aa sites 619, 2157, 2341, and 2502.

The interaction between FKBP12 and RyR1 alters DHPR-RyR1 functional interaction [11]. FKBP12 prevents leaky RYR1 signaling under sub-optimal ligand concentratations, and therefore serves as a molecular “gradient reader.” (Uniprot) Therefore, FKBPs are suggested to have “a stabilizing effect on RyR channel function by lowering open probability and preventing subconductance state gating,” which leads to “fewer leaky RyR channels and fewer aberrant Ca^++^ release events (Venturi et al. 2014).” Originally, FKBP12 was thought to stabilize RyR1 in skeletal muscle whereas FKBP12.6 stabilized RyR2 in cardiac cells [62]. Additional studies showed RyR2 likely undergoes dual modulation by FKBP12 and 12.6, such that FKBP12.6 acts as an FKBP12 antagonist indirectly reducing RyR2 open probability and SR Ca^++^ release. Since FKBP12.6 affects skeletal muscle function, dual modulation of RyR1 is also thought to occur [147].

The role of FKBP12 in the RyR1 EC-coupling pathway is controversial (Avila et al. 2003). Researchers initially demonstrated FKBP12 functions to close the RyR1 channel, but later showed FKBP12 also potentiates the RyR1 open state. According to Gaburjakova et al. (2001), the increased RyR1 gating frequency in the absence of FKBP12 suggests that FKBP12 functions to stabilize RyR1 in its open and closed states.

In group 1, the interaction between DHPR and RyR1 is modulated by FKBP12 [98] such that FKBP12 strongly potentiates the open state of RyR1 when RyR1 is bound to the Thr^671^-Lys^690^ region of the DHPR II-III loop [100]. In support, when FKBP12 was depleted, this resulted in obliteration of the open state of RyR1 following DHPR activation [100]. Not only does FKBP12 directly interact with RyR1 in the presence of DHPR activation, but it has also been identified in a group including RyR1, protein kinase A (PKA), phosphodiesterase 4D3 (PDE4D3), and protein phosphatase 1 (PP1). Within this group, FKBP12 functions to close the RyR1 channel, which is discussed in more detail later.

Given that FKBP12 plays a role in RyR1 open and closed states, future studies are required to determine if FKBP12 function changes depending on the RyR1 aa position to which FKBP12 binds. It may also be important for treatment purposes. For example, rycals, drugs that enhance FKBP12 binding to RyR1 [20], may be an effective treatment in the *RYR1*-RM population with mutations that negatively affect FKBP12 interaction with RyR1 and result in *RYR1*-RM. For this reason, we are currently performing a pre-clinical study testing the effect of Rycals on RyR1 function in muscle fibers biopsied from patients with *RYR1*-RM in collaboration with Marks and colleagues (unpublished data).

### Triadin

FKBP12 modulation of RyR1 activity is proposed to mediate the regulatory role of triadin on RyR1 activity [24]. Triadin is a junctional SR membrane glycoprotein that has been shown to interact with DHPR and RyR1 in the sarcoplasm [58, 68] supporting triadin playing a role in orthograde coupling. Disruption in RyR1 and triadin binding reduces orthograde signaling. However, it does not affect retrograde signaling between RyR1 and DHPR [67].

When the interaction between sarcoplasmic RyR1 and triadin is disrupted, this results in RyR1 channel inhibition. Amino acids 18–46 of triadin interact with RyR1 in the sarcoplasm at low Ca^++^ levels, whereas this binding is inhibited at high Ca^++^ levels. The prevention of amino acids 2–17 of triadin from binding to RyR1 by use of antibodies does affect RyR1 channel function, thereby resulting in a reduced rate of SR Ca^++^ release and decreased open probability of RyR1 [68].

Although triadin is not well understood, it is thought to regulate RyR1-DHPR interaction, and in turn, modulate EC coupling [53]. A number of studies have identified that triadin is “primarily a negative regulator of RyR1 [52]” [68, 70, 103, 131]. One study showed that amino acids 664 to 799 of DHPR alpha 1 subunit bind to triadin primarily at amino acids 68–278 [58]. Although triadin interacts with DHPR in the sarcoplasm, EC coupling and RyR1 channel regulation were not prevented in triadin null mice compared to wild type [131], yet, a significant reduction in muscle strength was shown in triadin null mice [101]. Interestingly, Shen et al. (2007) demonstrated little to no difference in force generation between wild type and triadin null mice in response to electrical stimulation. It is important to note that both Shen et al. (2007) and Oddoux et al. (2009) used triadin null mice, but electrically stimulated different muscles including the lumbricals and flexor digitorum of the hindlimb, respectively. These muscles may be affected differently in triadin null mice, and so affected muscle groups should be determined.

Despite no change in force generation, Shen et al. (2007) demonstrated electrical stimulation still resulted in a lower magnitude of Ca^++^ transients. Similar findings were shown in isolated myotubes from the same mice as well as a significant increase in resting myoplasmic Ca^++^ [131]. In support, Eltit et al. (2010) demonstrated chronically elevated resting myoplasmic Ca^++^ due to FKBP12-RyR1 dysfunction and SR store-operated Ca^++^ entry [52, 53], suggesting increased basal RyR1 activity in triadin null myotubes isolated from skeletal muscle of mice. Additionally, Oddoux et al. (2009) revealed a reduction of SR Ca^++^. Together, the results reveal that triadin ablation affects resting Ca^++^ levels such that there is an increase in myoplasmic Ca^++^ and a reduction in SR Ca^++^, supporting a lower magnitude of Ca^++^ transients in response to electrical stimulation. Eltit et al. (2011) also showed that triadin null mice result in no significant disruption of EC coupling. However, further kinetic analysis, in isolated myotubes, revealed that voltage-gated activation time for Ca^++^ release was slowed [53].

Given that triadin null mice presented with what appears to be a “normal” phenotype, it was suggested by Shen et al. (2007) that the role triadin plays in muscle function is minor or replaced with a compensatory mechanism [131] Conversely, according to Oddoux et al. (2009), a decrease in muscle strength in triadin null mice, suggests triadin dysfunction may lead to the development of a myopathy and is therefore essential for skeletal muscle function. Since both authors make valid points, whether or not the absence of triadin results in a RyR1 myopathy has yet to be determined.

Group 1 Pathomechanisms:

- **CCD/MH:** Mutations in the RyR1 N-terminal and central domains that disrupt FKBP12-RyR1 interaction are proposed to result in MH [65, 104] or CCD/MH [104]. V2461G and V2461I are RyR1 mutations that disrupt FKBP12 binding. In skeletal myotubes expressing these mutations, there was approximately a 50 % reduction in voltage-gated Ca + release due to the V2461G mutation compared to wild type. Myotubes expressing the V2461I mutation resulted in the binding of FKBP12.6 as opposed to FKBP12. In response, there was a reduction in SR Ca^++^ release [11]. A study performed by Galfre et al. (2013) identified three FKBP12 sites that interact with RyR1. Mutations introduced at any of these sites changed the function of FKBP12 such that mutant FKBP12 (at sites Glu31, Asp32, orTrp59) functioned as FKBP12.6, thereby activating the RyR1 channel and resulting in Ca^++^release. Based upon the above results, FKBP12 should be studied in more detail under different conditions.
- **CCRD:** Similar to I4898T, a well known mutation mentioned in group 3, the Y4796C mutation in RyR1 is also suspected to interfere with the interaction between RyR1 and triadin, but rather in the myoplasmic domain instead of the SR luminal region. Patients with the Y4796C mutation present with cores and rods on muscle biopsy, and therefore have CCRD. Consequently, there is increased rate of calcium leakage from the SR [95]. Y4637A and Y4637I mutations, like Y4796, also result in CCRD [90]. Amino acid 4637 is located in the membranous region of the RyR1 C-terminus. Similar to characteristics of the I4898T mutation, resting calcium levels associated with T4637A significantly increase and SR luminal Ca^++^ decreases. However, instead of leaky RyR1 channels as noted in CCD, individuals with the T4637A mutation present with excess ryanodine receptors in the central cores [127]. The T4637 pathomechanism may be the same for the Y4796C mutation rather than an increased rate of calcium leakage, but further research is needed.
- **CFTD:** Mutations associated with autosomal recessive myopathies often include a missense mutation along with a null mutation, and sometimes a homozygous missense mutation [10]. In a study with six patients diagnosed with CFTD, each patient exhibited a heterozygous missense mutation in addition a null mutation [37]. RYR1 mutations resulting in CFTD are linked to the RyR1-DHPR (α and β) binding sites.

#### Group 2

Group 2 (Fig. 1b) is located in the pore-forming region of RyR1 and is comprised of RyR1 interdomain interaction and calmodulin (CaM). This group responds to the activation of RyR1 by DHPR and is regulated by SR Ca^++^. It has been suggested that RyR1 conformational change in response to DHPR activation leads to RyR1 intrinsic modulation of the opening/closing of the RyR1 ion channel. This intrinsic modulation is based on inter-domain interaction where the RyR1 central domain (Leu^2442^-Pro^2477^) interacts with the RyR1 N-terminal domain [104]. This interdomain interaction is regulated by CaM and Ca^++^ levels regulate the function of CaM.

### RyR1 Interdomain interaction

Bannister et al. (2007) proposed the “domain switch” hypothesis that reflects the structure-function relationship between the interdomain interaction and RyR1 function. The hypothesis states that “In the non-activated state, the N-terminal and central domain make close contact through several sub-domains: this ‘zipped’ state stabilizes the closed state of the channel. Under normal stimulating conditions, the inter-domain contact is weakened leading to an ‘unzipped’ state, which is recognized by the channel as an activation signal.” Interestingly, MH mutations have been shown to result in a partial unzipped state leading to “hyperactivation/hypersensitization” of RyR1 [12].

Domain peptide 4 (DP4) is a synthetic peptide that corresponds to Leu^2442^-Pro^2477^ of RyR1. When DP4 was bound to the N-terminus of RyR1, this interaction resulted in an “unzipped” state that led to activation of ryanodine binding and SR Ca^++^ release [12]. Olojo et al. (2011) determined how the interdomain interaction influences orthograde and retrograde signaling by using DP4. The results showed enhanced RyR1 orthograde Ca^++^ release without affecting the DHPR voltage sensor and mediated retrograde signaling that results in a RyR1 open state [104]. In summary, the RyR1 conformational change in response to DHPR activation results in the “unzipped” state where the interdomain interaction is weakened and is recognized by RyR1 as an activation signal leading to the release of Ca^++^ [12]. Under normal conditions, the central domain and N-terminus make close contact maintaining the “zipped” state of RyR1 thereby stabilizing the RyR1 closed state [12].

### Calmodulin

Under normal conditions, CaM disrupts the interdomain interaction [77]. CaM exists in two forms, without Ca^++^ (apocalmodulin, apoCaM) and Ca^++^ bound (Ca^++^-CaM). Both forms bind to RyR1 with Ca^++^-CaM having a greater binding affinity [157]. ApoCaM serves as an agonist resulting in the release of Ca^++^ at low sarcoplasmic [Ca^++^], whereas Ca^++^-CaM maintains the closed state of RyR1 at high sarcoplasmic [Ca^++^] [64, 73, 77, 92, 157]. CaM levels increase as sarcoplasmic Ca^++^ levels increase. More recently, researchers showed that activation of CaM results in CaM Kinase II (CaMKII) activation, which, in turn, phosphorylates RyR1, affecting skeletal muscle contractility. In summary, Ca^++^-CaM binds to RyR1 on the sarcoplasmic side inhibiting SR Ca^++^ release, and, while bound, CAMKII phosphorylates RyR1 [64].

CAMKII, and as discussed later, PKA, both phosphorylate RyR1 and are thus considered modulators of RyR1 activity. It is important to note that hyperphosphorylation of RyR1 by CAMKII or PKA results in FKBP12 disassociation, and consequently, a higher open probability of RyR1. Along this continuum, a higher RyR1 open probability due to hyperphosphorylation may affect skeletal muscle contractility under resting conditions where skeletal muscle contractility is decreased [64].

In 2002, O’Connell et al. demonstrated that the introduction of CaM binding sites (3624 and 3620) in dyspedic myotubes primarily regulates L-type channel currents for retrograde signaling compared with EC coupling for orthograde signaling [99]. The structure of CaM is comprised of two lobes, a N- and C-lobe where the C-lobe of Ca^++^-CaM binds at RyR1 sites 3614–3643 [77] and the N-lobe to 1975–1999. Both of these undergo interdomain interaction [157]. Specifically, the interdomain interaction includes disulfide bonds formed between cysteine residues that include 3635, 2000, and 2401 [157] of adjacent RyR1 subunits within a tetramer. The ApoCaM-binding domain of RyR1 (Lys3614-Asn3643) also interacts with RyR1 sites Cys4114-Asn4142. When bound, this leads to Ca^++^ release [63]. ApoCaM not only binds to aa 3614–3643, but also aa 3625–3644 [117].

Although ApoCaM and Ca^++^-CaM have opposing functions, both prevent oxidation-induced intersubunit cross-linking where disulfide bonds are formed between each RyR1 subunit leading to Ca^++^ release [72, 110]. Post-translational modifications of RyR1, group 5, are discussed later. It is postulated that CaM protects RyR1 from oxidative stress associated with strenuous exercise [28, 73].

Conversely, oxidation of RyR1 prevents the binding of CaM (both forms) to RyR1 at low [Ca^++^]. Nitric oxide (NO), which plays a role in redox reactions involving RyR1, not only blocks intersubunit disulfide bonds formed by oxidation but also prevents the binding of ApoCaM [72, 110]. These data suggest that NO regulates oxidation and ApoCaM activity, both of which promote the RyR1 open state. The unaffected Ca^++^-CaM by NO, when bound to RyR1, results in the RyR1 closed state. The redox reactions are discussed later.

In the nitrosylation subsection, the literature demonstrates NO has a high affinity for CaM such that CaM is required for nitrosylation to occur. Ca^++^-CaM bound RyR1 is unaffected at most sites, thereby protecting RyR1 from oxidation. Further research is necessary to determine what occurs in a hypernitrosylated state or when mutations are present in the Ca^++^-CaM binding site on RyR1. If such changes result in a myopathy or malignant hyperthermia phenotype, this research would open the door to potential treatments. Additionally, given that CaM is not only required for nitrosylation, but also activates downstream phosphorylation of RyR1, it is important to determine whether hyperphosphorylation and hypernitrosylation occur simultaneously and possibly contribute to disease severity.

Interestingly, increased levels of CaM not only activate CAMKII, but also calcineurin. Calcineurin is a phosphatase responsible for skeletal muscle satellite cell differentiation, which is important for skeletal muscle fiber regeneration after injury and skeletal muscle hypertrophy [64, 145]. Activation of calcineurin primarily influences slow twitch fiber hypertrophy. In mice, inhibition of calcineurin resulted in marked inflammation, fiber atrophy, presence of immature myotubes, and calcification in regenerating muscle compared with controls [122, 123]. Further research is required to understand the role of calcinuerin in RYR1-RMs. Targeting calcineurin may be a potential therapeutic treatment.

Group 2 Pathomechanisms:

- **MH:** When DP4 was isolated in skinned skeletal muscle fibers, it enhanced ryanodine binding and sensitized the release of SR Ca^++^ similar to what has been shown in MH pathology. It is believed that MH results from the disrupted interdomain interactions between DP4 and the N-terminus of RyR1 that result in destabilization of the RyR1 closed state [87].
- **MmD:** Mutations in RyR1 that manifest as MmD are dispersed throughout RyR1 primarily outside the hot spot regions. RyR1 mutations P3527S and V4849I cause an increase in sarcoplasmic resting Ca^++^ without depleting SR Ca^++^ stores [143, 144, 159]. V4849I is an interesting mutation linked to autosomal recessive CCD, which presents as MmD [59].
  The aforementioned mutations are located in the S100A1 and CaM binding sites. Researchers are continously learning more about S100A1, but it is believed that this S100A1 is responsible for linking RyR1 subunits. S100A1 is considered one of the most important ligands in cardiac muscle, possibly skeletal muscle, and is also responsible for Ca^++^ release at low [Ca^++^]. A single site on RyR1 binds both S100A1 and Ca^++^-Cam. The release of Ca^++^ at low [Ca^++^] contributes to muscle twitches, however, the same site is critical for inhibiting Ca^++^ release during “repeated or sustained activation by binding Ca^++^-CaM at higher [Ca^++^]. In this way, Ca^++^ is able to slow energy expenditure later in contraction [92, 148]. Other RyR1 mutations, R109W (also P109W) and M485W, occur simultaneously and some are intronic variants such as homozygous 14646 splicing variant resulting in a reduced number of RyR1 [143, 144, 159]. MmD patients with these mutations clinically present with ophthalmoplegia and muscle weakness.
  Ophthalmoplegia, in this patient population, is thought to be due to the absence of RyR3 compensation [159]. In support, results reported by Perez et al. (2005) suggest RyR1 and RyR3 together regulate skeletal muscle Ca^++^. Ophthalmoplegia is also suspected to be mutation specific or caused by mitochondrial dysfunction [128, 130]. Causal *RYR1* mutations are located outside the hotspot regions or include a malignant hyperthermia causing mutation accompanied by another mutation outside the hotspot regions. Two known mutation combinations include R3772W + E989G and R3772W + H283R. A previously reported R3772Q mutation caused a more severe phenotype including ptosis, facial weakness, myopathy, and MHS. MRI pathophysiological findings potentially responsible for ophthalmoplegia, ptosis, and facial weakness include thin hypoplastic intraorbital motor cranial nerves in addition to hypoplasia of the extraocular muscles. Interestingly, the optic nerve remained healthy and intact [130]. Given the eye is a high-energy demand organ, the extraocular muscles are comprised of several mitochondria. However, chronic oxidative damage results in mitochondrial instability yielding mitochondrial damage. Mitochondrial dysfunction, increased oxidative stress, and increased apoptosis are common causes of ophthalmologic disorders in the aging population [128]. Although mitochondrial-related extraocular muscle dysfunction has not been shown in *RYR1*-RM, this pathomechanism may be worth assessing in this patient population.
- **CNM:** RyR1 mutations that manifest as CNM occur in DHPR, CaM, and sometimes the triadin binding sites disrupting interdomain interaction [3]. RyR1-CaM interaction can be disrupted in an environment with high oxidant concentrations [110]. Associated mutations include Glu1909GlyfsX39, Met3081Thr, Val4842Met, 10348-6C > G (intronic), Ser1342Gly, Thr2787Ser, and 3381 + 1 G > A (intronic). To achieve protein reduction, it is suggested that the aforementioned mutations coexist with the intronic mutation 10348-6C > G, which further results in the production of another mutation, His3449ins33fsX54 [82, 154].

#### Group 3

Group 3 (Fig. 1c) is located in the SR and is comprised of RyR1, CSQ, triadin, and junctin. This group responds to the RyR1 interdomain interaction and is regulated by SR Ca^++^. In group 3, CSQ seems to be the primary protein of interest for RyR1 channel activity because it indirectly regulates RyR1 open and closed states depending on SR [Ca^++^]. CSQ, in its phosphorylated and dephosphorylated states, regulates RyR1 channel activity through its interaction with junctin and triadin. The phosphorylated and dephosphorylated states of CSQ seem to be a regulatory mechanism of the CSQ/junctin/triadin complex, and the CSQ/junctin/triadin complex regulates RyR1 activity from the SR.

Triadin seems to be the key communicator between orthograde and retrograde signaling following voltage-gated activation of RyR1. Like FKBP12, it functions to potentiate both the open and closed states of RyR1. Triadin may also be a target for potential treatment. In group 1, FKBP12.6 restored resting Ca^++^ levels by acting directly on RyR1. Boncompagni showed that the SR luminal content and cisternae volume were significantly altered in triadin null mice [24]. How FKBP12.6 affects SR content while restoring resting levels is still to be determined. More studies are required to focus on the mechanisms of action between groups 1 and 3 as well as the pathomechanisms that result from RyR1 mutations that interfere with sarcoplasmic and SR luminal triadin binding sites.

### CSQ

CSQ is a Ca^++^ storage glycoprotein located in the lumen of the SR, which functions to lower the amount of free Ca^++^ in the SR [79, 132, 142]. More recently, studies have shown that CSQ is not only a storage protein. CSQ also modulates RyR1 channel activity [132] and is primarily located in close proximity to RyR1 [142]. Previous research has shown that the amount of Ca^++^ released from the SR is dependent on the amount of Ca^++^ bound to CSQ [79]. When CSQ is partially bound, small amounts of Ca^++^ are released at a high rate constant, whereas when fully bound, Ca^++^ is released at a slow rate constant [79]. In support, CSQ has been shown to have a controlled inhibitory effect on RyR. In the absence of CSQ, Ca^++^ release increased by 10 fold. This effect was reversed after reintroducing CSQ [15]. Specifically, the intraluminal phosphorylation/dephosphorylation of CSQ controlled RyR1 channel activity in the presence of Ca^++^. When CSQ is dephosphorylated, Ca^++^is released from the SR, but when phosphorylated, Ca^++^-bound CSQ has no effect on RyR1 [142].

It is important to note that CSQ does not directly interact with RyR1. Rather, it interacts indirectly through junctin and triadin [17]. Using a DCAM probe and electron microscopy, Ikemoto et al. (1989) showed Ca^++^ bound CSQ undergoes a conformational change, subsequently binding to junctional face membrane (jfm) proteins later identified as junctin and triadin [17, 103]. It was also demonstrated that conformational changes in CSQ were coupled to conformational changes in RyR1; a conformational change in one was transmitted to the other [142].

Junctin and triadin are transmembrane anchoring proteins that form a stable quaternary group, including RyR1, junctin, triadin, and CSQ. CSQ binds to junctin and triadin under low Ca^++^ concentrations resulting in the closed state of RyR1 [152]. Beard et al. (2008) demonstrated that when CSQ is phosphorylated under low SR luminal Ca^++^ concentrations, CSQ binds to junctin only. This phosphorylated state of CSQ does not disrupt the ability of CSQ to maintain the closed state of RyR1 [16]. Rather, it enhances the Ca^++^ binding affinity to CSQ [17]. These results suggest that the inhibitory effect of CSQ on RyR1 activity is mediated by junctin when CSQ is in its phosphorylated state under low Ca^++^ concentrations [17]. Under physiological conditions of Ca^++^, neither the phosphorylated nor the dephosphorylated state affects the coupling of CSQ, junctin, and triadin [16]. CSQ, in its dephosphorylated state under low SR Ca^++^ conditions, binds only to triadin, in turn activating ryanodine receptors. High luminal Ca^++^, on the other hand, results in dissociation of the CSQ, triadin, and junctin group [16]. Whether CSQ has an inhibitory [13, 14, 150] or activation [84, 102] effect on RyR1 has been controversial.

In summary, CSQ and RyR1 have an indirect relationship by way of triadin and junctin. This relationship appears to depend on SR luminal [Ca^++^] as well as phosphorylation/dephosphorylation mechanisms. Low SR luminal Ca^++^ promotes the binding of CSQ to junctin and triadin. This results in the RyR1 closed state. The binding of CSQ to junctin and triadin changes when phosphorylation and dephosphorylation occur. Low SR luminal Ca^++^ with CSQ phosphorylation still results in the binding of CSQ to junctin only with no RyR1 activity. However, in its dephosphorylated state, CSQ binds to triadin only and this interaction leads to RyR1 activation (Fig. 1c). This RyR1 activation is inhibited by ryanodine binding and cannot be reversed with dephosphorylated CSQ. Since CSQ does not bind to triadin and junctin when SR luminal Ca^++^ is high, based on current knowledge, CSQ only communicates with RyR1 when SR luminal Ca^++^ levels are low.

### Triadin

In group 1, triadin was shown to bind to RyR1 and DHPR on the sarcoplasmic side potentiating voltage-gated RyR1 Ca^++^ release. Triadin also interacts with RyR1 and CSQ in the SR lumen (group 3) in a Ca^++^ dependent manner serving as a linker protein between RyR1 and CSQ [69, 119]. The sarcoplasmic region of RyR1 and triadin become disassociated when the SR luminal binding of these proteins are disrupted, but do not affect RyR1 channel function [67]. Beard [18] based on these results, SR Ca^++^ and group 3 seem to regulate the function of triadin in group 1.

Three regions of triadin are responsible for its localization at the membrane. These regions include the targeting region (TR) 1 (18–47, sarcoplasmic), TR2 (106–214), and TR3 (233–440, 441–729). At least two of these three regions are required for correct localization in the membrane. Binding regions for RyR1 have been identified in TR3, and the same is true for CSQ [30]. Specifically, triadin binds to the SR luminal side of RyR1 at amino acids D4907, E4908, and D4878 [89]. CSQ appears to be associated with triadin stabilization (reduced mobility) in the SR membrane, and more importantly, a key component for the formation of a stable group between triadin and RyR1 [120].

Unlike the effect of triadin on RyR1 in group 1, triadin in group 3 functions to close the RyR1 channel while enhancing the binding affinity of ryanodine to RyR1 [67]. Ryanodine binding is used to study Ca^++^ binding affinity because ryanodine binding is Ca^++^ dependent. “Low affinity Ca^++^ binding sites resulted in inhibition of ryanodine binding and Ca^++^ release from isolated SR vesicles.” [71] Ohkura et al. (1998) showed that depletion of triadin increases ryanodine binding, but when available, triadin functions to inhibit ryanodine binding to the SR and maintain the closed state of RYR1. The effect of triadin on ryanodine binding is the same even when ryanodine binding is potentiated by CSQ [103].

Wei et al. [153] demonstrated that when triadin and junctin are exposed to RyR1 independently, the open state of RyR1 is enhanced. Once CSQ was added to each solution, only the RyR1/junctin interaction led to a reduction in RyR1 activity when the SR luminal Ca^++^ was lowered [18].

### Junctin

Junctin, like triadin, is a transmembrane protein that binds to RyR1 and CSQ. Unlike triadin, junctin only binds to the luminal domain of RyR1. Junctin is believed to play a more critical role in maintaining the SR Ca^++^ store and CSQ/RyR1 signaling in myotubes (Boncompagni et al. 2012-refs 38 and 39). Boncompagni et al. (2012) studied the function of triadin and junctin in Ca^++^ homeostasis using hind legs from mice (triadin null, junctin null, triadin/junctin null). Their results showed reduced coupling in triadin-null mice, whereas junctin null mice demonstrated minimal to no changes in functional activity. Based on these results, the interaction between triadin and CSQ has a major impact on the SR architecture and myoplasmic Ca^++^ [24] as previously noted in group 1. More specifically, the SR luminal content and volume of SR cisternae are significantly altered in triadin null and triadin/junctin null mice. CSQ is also less defined. The findings from Boncompagni et al. (2012) further support triadin as an important factor in skeletal muscle function as suggested in group 1.

Group 3 Pathomechanisms:

The triadin binding-domain in RyR1 is located within the hotspot 3 region of which gives rise to CCD and CCD/MH mutations [78]. Several *RYR1* mutations, resulting in central core disease, lead to amino acid changes in the SR luminal side of RyR1 that disrupt RyR1-triadin interaction as well as influence voltage-gated Ca^++^ release [11, 67]. I4898T is a very common RyR1 mutation within this SR luminal region that results in severe CCD and is proposed to possibly disrupt the interaction between RyR1 and triadin [95].

#### Group 4

Group 4, represented in Fig. 1d, responds to changes in adenosine 3’, 5’ cyclic monophosphate (cAMP), which is elevated due to acetylcholine (ACh) release. Group 1 responds to an action potential resulting from ACh release, whereas group 4 responds to elevated cAMP levels resulting from ACh release. Therefore, these groups may be activated simultaneously. Group 4 includes FKBP12, protein kinase A (PKA), phosphodiesterase 4D3 (PDE4D3), and protein phosphatase 1 (PP1). RyR1 undergoes phosphorylation and dephosphorylation [56] within the group.

cAMP levels are increased in response to acetylcholine release. As a result, PKA is activated and is anchored to RyR1 by way of A-kinase anchoring proteins of the skeletal muscle (mAKAP). PKA then phosphorylates RyR1 (S2483) preventing the binding of Mg^2+^ to RyR1, resulting in RyR1 open probability. PKA not only phosphorylates RyR1, but also phosphorylates the targeting subunit Gm, which results in the dissociation of PP1 from Gm and the SR. PP1 dissociation from Gm prevents PP1 from dephosphorylating RyR1. The above pathway results in RyR1 open probability. However, when cAMP levels are lowered by PDE4D3, PP1 is not dissociated from the Gm subunit nor is the Gm subunit separated from RyR1. PP1 is then able to dephosphorylate RyR1 resulting in FKBP12 binding, thus a RyR1 closed state. To better understand this portion of the pathway, the involved proteins of this group are further discussed.

Group 4 has several regulatory components and appears to be the only group that not only affects voltage-activated RyR1, but also adjacent non voltage-activated RyR1. Because group 4 affects both voltage-activated and non voltage-activated RyR1, this raises the question of whether mutations in RyR1 affecting this group influence clinical severity. Several of the aforementioned studies focused on a single component of the group, therefore, studies are needed that demonstrate pathomechanisms related to all components making up this group and their associated phenotype. For example, FKBP12 is responsible for synchronizing gating mechanisms between adjacent RyR1 proteins. Will a mutation affecting FKBP12 binding affinity to RyR1 and its ability to synchronize adjacent RyR1 influence severity? Additionally, as mentioned above, PKA phosphorylation and S-nitrosylation both dissociate FKBP12 from RyR1. What happens in skeletal muscle if the phosphorylation site on RyR1 is changed due to a mutation or hypernitrosylation? Lastly, in group 1, rycals were mentioned as a drug that enhances the binding affinity of FKBP12 to RyR1. Could this be a potential treatment that resolves any issue resulting from RyR1 mutations in this group? Further research is needed regarding group 4 and its related pathomechanisms.

### FKBP12

FKBP12 is not only a component of group 1, but also a component of group 4. In group 4, FKBP12 is an important regulatory protein. When bound to RyR1, it stabilizes the RyR1 closed state and synchronizes the gating between neighboring RyRs [39, 43, 147]. Neighboring RyR1 channels are very close to each other and are modulated by extracellular ligands, including Ca^++^, Mg^2+^, and ATP. Non-voltage activated neighboring RyR1 channels are activated via RyR1-RyR1 physical interaction, and are stabilized by luminal Ca^++^ and cytosolic ATP/Mg^2+^ [109].

FKBP12 is suggested to coordinate this multiprotein group formation [114] such that bound FKBP12 does not promote RyR1 activity. However, FKBP12 dissociates from RyR1 due to PKA phosphorylation at RyR1 sites S2843 in humans [19, 114] and S2844 in mice [19, 114], yet becomes bound again due to PP1 activity. PKA and PP1 functions are discussed below.

Similar to PKA phosphorylation, S-nitrosylation (group 5) of RyR1 also reduces the binding affinity of FKBP12 to RyR1; specifically, S-nitrosylation of cysteine residues at positions 3635 and 2327 [7, 8, 19, 136]. Unlike PKA, S-nitrosylation does not respond to cAMP as discussed later under group 5.

### PKA

PKA is a holoenzyme with a tetrameric group consisting of two catalytic (C) subunits and a regulatory subunit dimer. Adrenaline, a hormone that acts on the skeletal muscle in response to neural ACh release [91], elevates cAMP levels resulting in PKA activation, which in turn induces Gm phosphorylation [149].

cAMP is required for PKA phosphorylation of the RyR1 channel, otherwise referred to as cAMP-induced PKA phosphorylation [114]. When cAMP levels are low, the C subunit binds to the regulatory subunit making PKA inactive. On the other hand, in the presence of high levels of cAMP, cAMP binds to the regulatory C subunit. In turn, the affinity of the regulatory subunit for the C subunit is reduced thus freeing the C subunits and activating PKA [34]. mAKAPs co-localize with RyR1 and function to anchor PKA to RyR1 in the presence of elevated cAMP levels [121]. In response, PKA phosphorylates serine residue, S2843 [19, 114], on RyR1 subunits in the sarcoplasm leading to a skeletal muscle contraction and greater muscle force generation [5]. PKA-dependent phosphorylation prevents the binding of Mg^2+^ to the RyR1 channel thereby increasing RyR1 open probability [121].

Additionally, glutathionylation regulates PKA activity, which regulates RyR1 activity in the presence of cAMP. PKA cannot be glutathionylated in the absence of cAMP and is therefore protected from oxidation. In the presence of cAMP, PKA becomes active. Once active, glutathionylation makes PKA more susceptible to dephosphorylation, and thus its’ inhibition. “PKA deglutathionylation leads to PKA reactivation” [106].

### PDE4D3

Like PKA, PDE4D3 is targeted to RyR1 by way of mAKAP, which is an anchoring protein [19]. PDE4D3 is specific for cAMP [28]. Phosphodiesterases regulate cAMP levels by binding and degrading cAMP. PDE4D3, specifically, functions to control cAMP concentration by degradation when co-localized with RyR1 [19].

### PP1

PP1 dephosphorylates RyR1 [114] resulting in the binding of FKBP12 to RyR1. PP1 is a serine/threonine kinase with a catalytic subunit and several targeting subunits. Specifically, the Gm targeting subunit of PP1 binds and directs PP1 to glycogen particles and the SR. PP1 binds to the Gm N-terminus and the SR to its C-terminus. However, phosphorylation of Gm at Ser67, by PKA, dissociates PP1 from the Gm binding domain subsequently releasing Gm from both glycogen and the SR [149].

#### Group 5

Group 5, shown in Fig. 1e, responds to changes in muscle O_2_ and glutathione ratio (GSH/GSSG). This group encompasses protein post-translational modifications including S-nitrosylation, S-oxidation, S-glutathionylation, and S-palmitoylation [56] and the molecules nitric oxide (NO), S-nitrosoglutathione (GSNO), reduced glutathione (GSH), oxidized glutathione (GSSG), hydrogen peroxide (H_2_O_2_).

Within this group, RyR1 serves as a redox sensor where certain cysteine residues undergo redox reactions by way of post-translational modifications [7]. RyR1, as a redox sensor, is enhanced by the aforementioned molecules [9]. Each of these post-translational modifications occur based on O_2_ levels, which change depending on oxygen demand of the active muscle [139].

Post-translational modifications serve as on/off switches of protein function [96]. S-nitrosylation, S-oxidation (disulfide oxidation), and S-glutathionylation each activate RyR1 by way of different mechanisms [9]. However, together, they regulate RyR1 channel activity over a range of skeletal muscle oxygen tension (pO_2_) [141].

### S-nitrosylation

Physiological pO_2_ levels (~4–20 mm Hg, 0.5-2.5 %) control the redox state of thiols in the RyR1 subunits maintaining the ready state of RyR1. NO, at physiological tissue pO_2_ (~10 mm Hg), activates RyR1 by S-nitrosylation of RyR1 cysteine residues. Both reactive oxygen and nitrogen species modify RyR1 thiols altering RyR1 channel function [56, 57]. Oxidation and nitrosylation enhance Ca^++^ release from the SR via the RyR1 channel [141].

In skeletal muscle, NO is derived from neuronal NO synthase (nNOS) and functions to S-nitrosylate proteins forming S-nitrosothiols [139]. S-nitrosothiols are compounds that S-nitrosate a specific protein cys thiol [25]. S-nitrosoglutathione (GSNO), formed by NO and GSH interaction, is an example of a nitrosothiol [25]. GSNO, under atmospheric pO_2_, GSNO can nitrosylate and glutathionylate RyR1 cysteine residues [9]. Specifically, in vitro GSNO treatment resulted in nitrosylation of RyR1 aa residues 1–1509 while decreasing S-nitrosylation at residues 3120–4475 and 3631–4475. Glutathionylation occurred at the same residues, further including aa 1396–2401. Although GSNO is able to both nitrosylate and glutathionylate RyR1, glutathionylation seems to be preferred [9]. GSNO is the S-nitrosated derivative of glutathione and is considered to be a pertinent mediator of NO. It is the intermediate in the formation and degradation of S-nitrosothiols, and for this reason, it is considered to be potentially therapeutic [25]. GSNO not only activates RyR1 by nitrosylation, but also oxidation (C. Hidalgo 2005).

It is important to note that NO only nitrosylates RyR1 cysteine residues in the presence of CaM and at low muscle pO_2_ levels [136, 139]. Specifically, in the skeletal muscle, 6–8 RyR1 thiols are S-nitrosylated [36, 135]. Cys3635 is one of the 6–8 residues identified that is nitrosylated at low pO_2_, but not at high pO_2_ [141]. Consequently, RyR1 changes conformation to the open state promoting Ca^++^ release and muscle force production [135]. Interestingly, Cys3635 is one cysteine residue that is unaffected by GSNO for Cys3635 does not discriminate between O_2_ levels [75].

Eu et al. (2000) determined that the effect of NO on RyR activity is dependent on the RyR1 redox state as well as CaM. Brookes et al. (2004) believe mitochondria may serve as a “redox signaling box” by converting the NO signal into an ROS signal [26, 27]. This phenomenon only occurs at physiological levels of NO. In skeletal muscle, NO is produced by nitric oxide synthases (NOS) in the sarcolemma and muscular endplate [60] to maintain skeletal muscle response to increased exercise. It is important to note that NOS activity inhibits mitochondrial respiration [136]. More specifically, NO in the presence of high [Ca^++^] inhibits mitochondrial respiration [27, 133]. However, pathological levels of NO disrupt this process, affecting mitochondrial function, and, in turn, ATP synthesis and cell function [27, 133].

It has been shown that NO in skeletal muscle is produced at rest and in greater concentrations with increased exercise. In addition to the increase in NO, there is also an increase in reactive oxygen and nitrogen species due to increased muscle contractile activity. In response to exercise, NOS binds CaM, which enhances NOS activity. CaM serves as a molecular switch activating the transfer of electrons that results in NO production [138]. Although CaM plays such a major role in S-nitrosylation, RyR1-bound Ca^++^-CaM is left unaffected during the process [56, 75]. However, Ca^++^-CaM bound RyR1 at site Cys3635 (to date, it is the only site known to date) is affected by S-nitrosylation, reversing its inhibitory effect and resulting in RyR1 activation [75, 141].

In summary, NO seems to regulate oxidative and glycolytic activity in skeletal muscle, which is further discussed in the “oxidative stress” section below. NO functions to activate RyR1 in the presence of CaM and low O_2_ levels. It also functions to inhibit mitochondrial respiration in the presence of high [Ca^++^]. On the other hand, at physiological NO, mitochondria convert the NO signal into a redox signal yielding reactive nitrogen and oxygen species. The dominant form of RyR1 myopathies manifests clinically due to a leaky RyR1 channel, which results in excessive skeletal muscle Ca^++^. Future research in RyR1 myopathies should not only observe Ca^++^ regulation with different RyR1 mutations but also NOS activity and localization, NO levels, CaM levels and CaM-bound NOS together. Excessive Ca^++^, theoretically, would deplete NO, and in turn reduce the frequency of inhibition of mitochondrial respiration. Consequently, this could result in excessive production of RNS and ROS via mitochondrial respiration. If this is the case, treatment targeting NO signaling may be beneficial in this patient population.

### S-oxidation

S-oxidation is coupled to S-nitrosylation. As muscle O_2_ levels change, there is a transition from nitrosylation to oxidation and visa versa. “O_2_ based signaling is mediated by reversible RyR1 channel oxidation/reduction coupled to H_2_O_2_ production by SR-resident NADPH oxidase 4 (Nox4) that results in channel activation/deactivation [140, 141].” Nox4 is considered an O_2_ sensor in skeletal muscle. (Sun et al. 2011) Reactive oxygen species (ROS, superoxide anions and H_2_O_2_) are oxidizing molecules produced by Nox4 [76]. They are generated in proportion to pO_2_ in the SR [141].

More specifically, S-oxidation of RyR1 is determined by muscle pO_2_ where there is an O_2_-dependent production of H_2_O_2_ by Nox4, and so oxidation primarily occurs at high O_2_ concentrations [141]. H_2_O_2_ are reactive oxygen species that oxidize the RyR1 cys-thiols. Like NO, ROS activate the RyR1 channel; releasing Ca^++^ from the SR.

Oxidants activate RyR1 by producing inter-subunit disulfide linkages [72, 110] whereas CaM-bound RyR1 (ApoCaM and Ca^++^-CaM) prevents the formation of the intersubunit disulfide linkages. Conversely, CaM interaction with RyR1 is inhibited by oxidation [110]. Hamilton [72] cys3635 has been identified as an inter-subunit contact site and is located within the CaM binding region [72]. Moore et al. (1999) identified one CaM binding site per RyR1 subunit at high or low Ca^++^ levels. Interestingly, a mutation at Cys3635 does not interfere with RyR1 activation by the oxidizing molecule H_2_O_2_, as it is not required for RyR1 to serve as a redox sensor [9]. Essentially, CaM protects RyR1 from oxidation. However, high concentrations of oxidants (oxidative stress) may result in the loss of RyR1 bound CaM during which a person may experience fatigue [110].

Cysteine residues that are coupled to muscle oxygen tension are located in the cytoplasmic domain of RyR1 and regulate RyR1 interaction with DHPR and FKBP12. Other cysteine residues are located in hotspot regions that correspond to different diseased states including MH and CCD. The residues within the hotspot regions undergo oxidation, but not glutathionylation. Yet, glutathionylation is a reversible oxidative modification [141].

### S-glutathionylation

During physical exercise, endogenous glutathione is modulated with high oxygen consumption and ROS generation [111]. Although not well understood, the glutathione ratio dictates cellular redox potential [42, 94]. Physiologically, the sarcoplasm is a reducing environment in which the protein redox state is dependent on the GSH/GSSG ratio, and a high GSH/GSSG ratio in the cytosol creates a redox buffer [42]. A GSH/GSSG ratio above 100 promotes s-glutathionylation, as does the oxidation of small amounts of GSH [42, 94]. S-glutathionylation of RyR1 functions to decrease RyR1 sensitivity to Mg^2+^inhibition maintaining RyR1 open probability [7, 8].

To date, researchers have identified the superoxide anion, H_2_O_2_, as a primary oxidizing molecule for RyR1 glutathionylation even though oxidized glutathione (GSSG) and O_2_ have also been shown to enhance RyR1 channel activity. H_2_O_2_, in the presence of reduced glutathione (GSH), reacts with redox sensitive RyR1 for glutathionylation of RyR1 as well as enhances the process [7, 75].

Specifically, redox-sensitive proteins, like RyR1, have cysteine residues that exist as thiolate anions at neutral pH, unlike proteins that are not redox-sensitive. Proteins that are not redox sensitive have cysteine residues that remain protonated. The difference in charge between cysteine residues that are thiolate anions and cysteine residues that are protonated, within a redox-sensitive protein, make the thiolate anions “active cysteines” that are vulnerable to oxidation. H_2_O_2_ oxidizes the protein thiols creating an unstable protein sulfenic acid that serves as an intermediate. The sulfenic acid then undergoes glutathionylation during which they become thiolated forming a disulfide bond with GSH [42, 106].

In the presence of oxidative stress, proteins are targeted for s-glutathionylation. GSH then becomes depleted and there is an increase in the oxidized derivatives (GS-, GSNO, and GSSG) resulting in a decreased GSH/GSSG ratio (Mieyal et al. 2008). It is important to note that both GSNO and GSSG, in addition to GSH, are responsible for protein glutathionylation. Consequently, under stressful conditions, these factors may be responsible for the development of pathological states through the stimulation of uncontrolled calcium-induced calcium release [8]. Upon restoration of the GSH/GSSG ratio, S-glutathionylation is reversed [42, 49]. Durham et al. (2008) inhibited NOS in mutant mice, and in doing so, restored the GSH/GSSG ratio. T-tubule NOS has been shown to promote RyR1 glutathionylation [106]. Under physiological conditions, NO levels derived from nNOS are lower than the GSH/GSSG ratio; however, when nNOS-related NO production increases, the enzymes responsible for glutathione synthesis are inhibited. The Durham results suggest that nitrosative stress mediates oxidative stress and that GSH/GSSG ratio is decreased in Y522S mouse models [50, 137]. N-acetylcysteine is a precursor of glutathione and successfully restored GSH/GSSG ratio in this model. Hind limb muscle force in the mouse also improved with NAC [50].

### S-palmitoylation

Palmitoylation is a reversible process where the 16-carbon saturated fatty acid palmitate forms a thioester link to cysteine thiols creating an acyl chain [23]. Removing palmitate from RyR1 diminishes RyR1 Ca^++^ release. S-palmitoylation includes the modification of at least 18 RyR1 cys residues. These residues have been identified in protein interaction regions for DHPR, CaM, and FKBP12. They are also located in RyR1 hot spot regions that correspond to malignant hyperthermia and central core disease. Eight of the 18 residues are cys residues that are also subject to S-nitrosylation and S-glutathionylation [33].

Palmitoylation is one of the least studied processes in the RyR1 pathway, yet may be a potential treatment. Palmitoylation removes palmitate from two binding sites (CaM and FKBP12), of which may function to close the RyR1 channel. It would be interesting to study the differences in palmitate levels in RyR1 myopathic muscle compared with “normal” tissue.

Group 5 Pathomechanisms:

- **CCD/MH:** Cysteine residues at sites 36, 253, and 315 are located in hot spot 1 of RyR1 whereas Cys residues between 2326 and 2363 are located in hot spot 2. Mutations at these sites interfere with regulation of RyR1 resulting in MH [9].
  Hyper-S-nitrosylation of RyR1 results in FKBP12 depletion thus leaky channels in muscular dystrophin mice. Together, hyper-S-nitrosylation and FKBP12 depletion are suggested to contribute to muscle weakness in muscular dystrophy [20, 74]. This may also be the case for muscle weakness observed in individuals with RyR1 myopathies as discussed above under CCD and S-nitrosylation.
  The Y522S mutation in RyR1, although not located in the RyR1-DHPR binding site, alters DHPR inactivation during retrograde signaling where there is an increase in Ca^++^ release [6]. This mutation is located in hotspot 1 and results in CCD/MH.
  Durham et al. (2008) studied S-nitrosylation in RyR1 mice with a point mutation (Y522S) associated with MH, and in humans, central core disease. The Y522S mutation resulted in RyR1 Ca^++^ leak that led to increased production of reactive nitrogen species (RNS). S-nitrosylation, following excessive RNS production, results in “increased temperature sensitivity for RyR1 activation, producing muscle contractures upon exposure to elevated temperatures.” Additionally, the mitochondria are abnormally shaped, there is increased mitochondrial lipid peroxidation, and decreased muscle force production [50]. In Y522S knock-in mice, there were elevated ROS, leaky channels, and damaged enlarged mitochondria [51]. Following N-acetylcysteine administration, the mitochondria and muscles were protected against oxidative damage and reduced force production, respectively [50].
  Similar to Y522S, R163C knock-in mice also presented with greater sarcoplasmic [Ca^++^] and ROS, but rather a different pathomechanism and manifests as MH only. In myotubes of R163C knock-in mice, the Ca^++^ decay rate is slowed such that the RyR1 retrograde signal is altered thus resulting in delayed DHPR inactivation. It is important to note that MH due to the R163C mutant does not result in SR Ca^++^ depletion or RyR1 inactivation suggesting no leaky channels [55]. Another study of the R163C mutant mouse, showed increased mitochondrial Ca^++^ and ROS as well as reduced oxidative phosphorylation and lower mitochondrial protein expression. Ultimately, the R163C mutation results in elevated sarcoplasmic resting Ca^++^ levels [51, 66]
  Abnormal oxidation of RyR1 cys thiols may be connected to dysregulation of S-nitrosylation, of which leads to an RyR1 Ca^++^ leak resulting in various muscle pathologies, including exercise-induced fatigue, CCD, and MH [9, 140]. RyR1 activation due to oxidation is prevented by NO, of which nitrosylation is CAM dependent [1, 113]. Conversely, RyR1 is activated by oxidation of RyR1 cys thiols at high PO^2^ concentration and this oxidation prevents S-nitrosylation of a separate cys thiol that activates RyR1 at low PO2 [56, 57, 140].
- **CCD or MH:** S-palmitoylation targets cysteine residues in the hot spot regions that are linked to MH and CCD and are interaction sites for DHPR, CaM, and FKBP12 [33]. The severity of one’s condition or onset of MH may be due to post-translational modifications at CCD/MH mutation sites.
- **Exercise Intolerance:** During exercise, RyR1 is progressively PKA-hyperphosphorylated, S-nitrosylated, and depleted of PDE4D3 and FKBP12 ultimately resulting in “leaky” channels thus decreased exercise tolerance. S107, a type of Rycal, prevents the depletion of FKBP12, and in turn improves force generation and exercise capacity [19]. FKBP12 dissociates from RyR1 due to PKA phosphorylation at RyR1 sites S2843 in humans [114] and S2844 in mice [19, 114]. However, a mutation (S2843A and S2844A) at these sites does not allow for PKA phosphorylation of the RyR1 channel and thus a decrease in the RyR1 open probability. S2843D and S2844D mutations, on the other hand, mimic PKA phosphorylation otherwise referred to as hyperphosphorylation, thus increasing RyR1 open probability [19, 114].

#### Group 6

Group 6 seems to respond to [Ca^++^]. This group includes three different ligands including calcium (Ca^++^), adenosine triphosphate (ATP), and magnesium (Mg^2+^), which are extracellular ligands that regulate RyR1 activity [56]. RyR1 includes two types of sites for ligand binding, activation and inhibitory sites. The activation sites are referred to as A-sites, whereas the inhibitory sites are called I-sites [92]. Ca^++^ and ATP bind to the RyR1 A-sites increasing RyR1 open probability, whereas Ca^++^ and Mg^2+^ bind to the I-sites promoting the RyR1 closed state [88, 92] I-sites are divalent, nonspecific cation sites to which both Ca^++^ and Mg^2+^ bind. The binding affinity of these cations to the I-sites are unaffected by ATP unlike the binding of activating Ca^++^ to the A-sites [88]. Figure 1f outlines the group 6.

### Ca^++^

Recently, RyR1 has been shown to have an alpha solenoid scaffold in the cytosolic region. In the core solenoid (starting at aa3679) of this region, there are calmodulin (CaM)-like binding domains referred to as the putative Ca^++^ binding domain and suggested to serve as Ca^++^ sensors. “Six of the eight residues that coordinate Ca^++^ in CaM are conserved in the putative Ca^++^-binding domain of RyR1. Since the S2 and S3 helices (aa4675-4790) are located close to putative Ca^++^ binding domains and the C-terminal, it is thought that they contribute to transmitting Ca^++^-mediated RyR1 conformational changes to the cytosolic formation of the pore [156].

Ca^++^ has both an activating and inhibitory effect on RyR1 when bound [88]. Physiologic Ca^++^ levels yield an un-stimulatory effect on RyR1 [54]. Upon skeletal muscle stimulation, Ca^++^ is released from the SR into the sarcoplasm triggering several downstream events [54]. As evidenced by RyR1 truncation experiments, Ca^++^ activation sites are suggested to be located between aa4007-5037, within the pore-forming region of the RyR1 cytoplasmic subunits. Ca^++^ activation sites on ryanodine receptors are located in the cytoplasmic region of ryanodine receptors and are referred to as A-sites. Specifically, aa4032 is a part of the “A-site gating-mechanism” as evidenced by the introduction of the E4032A mutation, which led to a significant decrease in sarcoplasmic Ca^++^ activation [88]. Amino acids 1873–1903, 1641–2437, and 615 have been implicated as the inhibitory sites.

### ATP

When bound to ATP at one or more of its ATP binding sites, RyR1 is activated. However, the interaction between ATP and RyR1 is affected by Ca^++^, Mg^2+^, and pharmacologic agents including dantrolene [44]. In the presence of activating Ca^++^, the ATP binding affinity decreases, whereas it increases in the presence of inhibitory Ca^++^. Although the ATP binding affinity increases in the presence of inhibitory Ca^++^, the number of ATP accessible sites decreases [44]. The different sites at which ATP binds has yet to be determined. Popova et al. (2012) identified four potential ATP binding sites on RyR1 including aa 699–704, 701–706, 1081–1084, and 1195–1200. These sites are located next to the RyR1 hotspot regions [108].

### Mg^2+^

In contrast to ATP, Mg^2+^ inhibits RyR1 activity. Mg^2+^ binds to both high affinity Ca^++^ activation sites as well Mg^2+^ inhibitory sites [38]. Although Mg^2+^ binds to the A-site, it does not activate the RyR1 channel [92]. Instead, it yields an inhibitory effect by reducing RyR1 sensitivity to Ca^++^ [54]. Under normal physiological conditions, Mg^2+^ remains bound to the RyR1 I-sites inhibiting the activation effect of both Ca^++^ and ATP [92].

## Conclusion

In this review, several studies were compiled to outline the RyR1 pathway and *RYR1*-RM-related pathomechanisms in an effort to highlight potential target sites for treatment as well as areas that require further exploration. Given the complexity of the pathway, we divided its interactions and modifications into six regulatory groups. FKBP12, Triadin, and CaM were the only identified interacting proteins that function across all six groups. Not only do they function across groups, but pathogenic mutations located at their RyR1 interaction sites also clinically present as CCD, MmD, and CNM [3, 65, 78, 104]. Additionally, post-translational modifications in the presence of mutations related to FKBP12 and CaM binding sites contribute to CCD/MH [19, 33, 50, 114]. Based on this information, these interaction sites serve as potential sites of treatment targets.

Evidently, post-translational modifications also play a major role in regulating the RyR1 channel. For example, nitric oxide nitrosylates RyR1 but also affects mitochondrial activity [50]. Additionally, nitrosylation in the presence certain RyR1 mutations, such as Y522S, increases temperature sensitivity of RyR1 activation to elevated temperatures. Thus further research related to hyper-nitrosylation and its role in disease severity is needed. Oxidation is coupled to nitrosylation such that both processes mediate the other [1, 9, 113, 140]. Phosphorylation affects FKBP12 in the sarcoplasm, CSQ activity in the SR, and PP1 activity that influences RyR1 function [19, 114]. Palmitoylation occurs at binding sites for FKBP12, CaM, and DHPR as well as at sites that are subject to nitrosylation and glutathionylation [33]. The interplay between different post-translational modifications may contribute to disease severity in the presence of pathogenic mutations.

Although much remains to be learned about the RyR1 pathway, the potential for effective treatments exists based on what is currently known. As mentioned previously, we are currently performing the first randomized, placebo-controlled, double-blinded drug trial in patients with *RYR1*-RM using N-acetylcysteine to target mitochondrial oxidative stress [48]. Additionally, pre-clinical studies are ongoing using rycals, which target FKBP12 binding affinity [4, 20], one of the three interacting proteins that functions across groups. Gene replacement therapy has also been considered for RYR1-RM but proves difficult due to the large size of the *RYR1* gene and the challenge of inserting the full gene into a delivery vector [115]. On the other hand, CRISPR/Cas9 technology offers promise by correcting the specific mutation in each patient and is currently in beginning stages in mouse models of RYR1-RM. As RyR1 plays a major role in skeletal muscle calcium regulation in general, components of these potential therapies may apply to RYR1-RM plus other conditions related to calcium dysregulation in the future.
